# Cuprous Oxide Nanoparticles: Synthesis, Characterization, and Their Application for Enhancing the Humidity-Sensing Properties of Poly(dioctylfluorene)

**DOI:** 10.3390/polym14081503

**Published:** 2022-04-07

**Authors:** Muhammad Tahir, Muhammad Zeb, Alamgeer, Shahid Hussain, Mahidur R. Sarker, Dil Nawaz Khan, Fazal Wahab, Sawal Hamid Md Ali

**Affiliations:** 1 Department of Physics, Faculty of Physical and Numerical Sciences, Abdul Wali Khan University Mardan, Mardan 23200, Pakistan; mzebphy@gmail.com (M.Z.); alamgeerphysics45@gmail.com (A.); shahidbalagarhi@gmail.com (S.H.); 2 Institute of IR 4.0, University Kebangsaan Malaysia, Bangi 43600, Malaysia; 3 Pak-Austria Fachhochschule—Institute of Applied Sciences and Technology, Haripur 22620, Pakistan; dilnawaz73@gmail.com; 4 Department of Physics, Karakoram International University, Gilgit 15100, Pakistan; fazal.wahab@kiu.edu.pk; 5 Department of Electrical, Electronic and Systems Engineering, Faculty of Engineering and Built Environment, University Kebangsaan Malaysia, Bangi 43600, Malaysia

**Keywords:** conducting polymer, poly(9,9 dioctylfluorene) (F8), Cu_2_O nanoparticles, humidity sensing, organic–inorganic nanocomposites

## Abstract

In this paper, we report on the synthesis—via the wet chemical precipitation route method—and thin film characteristics of inorganic semiconductor, cuprous oxide (Cu_2_O) nanoparticles, for their potential application in enhancing the humidity-sensing properties of semiconducting polymer poly(9,9-dioctylfluorene) (F8). For morphological analysis of the synthesized Cu_2_O nanoparticles, transmission electron microscope (TEM) and scanning electron microscope (SEM) micrographs are studied to investigate the texture, distribution, shape, and sizes of Cu_2_O crystallites. The TEM image of the Cu_2_O nanoparticles exhibits somewhat non-uniform distribution with almost uniform shape and size having an average particle size of ≈24 ± 2 nm. Fourier transformed infrared (FTIR) and X-ray diffraction (XRD) spectra are studied to validate the formation of Cu_2_O nanoparticles. Additionally, atomic force microscopy (AFM) is performed to analyze the surface morphology of polymer-inorganic (F8-Cu_2_O) nanocomposites thin film to see the grain sizes, mosaics, and average surface roughness. In order to study the enhancement in sensing properties of F8, a hybrid organic–inorganic (F8-Cu_2_O) surface-type humidity sensor Ag/F8-Cu_2_O/Ag is fabricated by employing F8 polymer as an active matrix layer and Cu_2_O nanoparticles as a dopant. The Ag/F8-Cu_2_O/Ag device is prepared by spin coating a 10:1 wt% solution of F8-Cu_2_O nanocomposite on pre-patterned silver (Ag) electrodes on glass. The inter-electrode gap (≈5 μm) between Ag is developed by photolithography. To study humidity sensing, the Ag/F8-Cu_2_O/Ag device is characterized by measuring its capacitance (C) as a function of relative humidity (%RH) at two different frequencies (120 Hz and 1 kHz). The device exhibits a broad humidity sensing range (27–86%RH) with shorter response time and recovery time, i.e., 9 s and 8 s, respectively. The present results show significant enhancement in the humidity-sensing properties as compared to our previously reported results of Ag/F8/Ag sensor wherein the humidity sensing range was 45–78%RH with 15 s and 7 s response and recovery times, respectively. The improvement in the humidity-sensing properties is attributed to the potential use of Cu_2_O nanoparticles, which change the hydrophobicity, surface to volume ratio of Cu_2_O nanoparticles, as well as modification in electron polarizability and polarity of the F8 matrix layer.

## 1. Introduction

The potential use of nanomaterials such as quantum dots, nanowires, nanosheets, and nanoparticles have brought significance revolution in electronic, optoelectronic, and drug delivery systems [1,2,3,4]. It is due to their exciting and extraordinary properties at the nanoscale, i.e., quantum confinements and large surface-to-volume ratio of the nanomaterials. Nanomaterials have been successfully employed in novel hybrid organic–inorganic nanocomposite devices such as photodetector sensors, solar cells, field effect transistors, etc. [5]. The main reason to synthesize nanomaterials is to tune the quantum mechanical properties which are not achievable in bulk materials. On the other hand, nanocomposites made of organic–inorganic nanoparticles have materials with synergetic behaviors such as exceptional electrical, chemical, optical and mechanical properties, which are unavailable in the one-component materials; they also have received remarkable attention for various device applications such as humidity and gas sensors [6,7].

Among organic semiconductors, conducting polymers are famous for their potential use in electronics, optoelectronics, and sensors. Polyfluorenes and its derivatives belong to a class of polymeric semiconductors that demonstrate comparatively higher charge carriers mobility as well as good chemical, mechanical and thermal stability [8]. Poly(9,9-dioctylfluorene) (F8) is a semiconducting polymer with the unique characteristics such as its solubility in several solvents, good π-conjugation structure with high hole mobility, large photoluminescence quantum efficiency, stability at higher humidity levels, and strongly hydrophobic behavior. F8 has been studied for its potential applications in mechanically flexible and low-cost semiconductor devices such as organic light-emitting diodes (OLEDs), organic field effect transistors (OFETs), humidity sensors, and temperature sensors [9]. These extensive applications are due to the interesting properties of F8 originating from its various chain conformations and multiple phase structures. One of the main advantages of the solution-processable conjugated polymers, such as F8, is that they allow making use of blending process to tune its properties where two and/or more polymers or some functional nanomaterials are mixed together in such way to obtain desirable properties of the mixture without the synthesis/chemical reaction of new materials [10]. This results in the production of such kind of materials and/or nanocomposites that possess novel structural, electronic, and optical properties that are different from their individual constituents [2].

Cuprous oxide (Cu_2_O) is an inorganic p-type semiconductor that possesses extraordinary electrical and optoelectronic properties with a narrow optical bandgap, abundant availability, non-toxicity, and low-cost [11]. The role of Cu_2_O nanoparticles is proven to tune/improve the electronic and optoelectronic properties of bulk materials in the form of its nanocomposites [12]. Previous studies show that Cu_2_O has exhibited significant enhancement in gas-sensing characteristics, biosensing, photocatalytic properties, and water-splitting of various inorganic host materials, i.e.,Cu_2_O–NaNbO_3_ [13], Cu_2_O–SnO_2_ [14,15], Cu_2_O–SnO [16], Cu_2_O–RuO_2_ [17], Cu_2_O–SiO_2_ [18], Cu_2_O-SrTiO_3_ [19,20], Cu_2_O–CeO_2_ [21,22], Cu_2_O–Co_3_O_4_ [23], etc. Hence, keeping in view the aforementioned applications, Cu_2_O nanoparticles can also possibly possess the capability to enhance humidity-sensing characteristics as well because of their hydrophobic nature, chemical as well as thermal stability, and large surface-to-volume ratio available for sensing. To exploit these properties, Cu_2_O may find suitable applications in sensors such as humidity, temperature, and pressure sensors. However, to date, there is no study available in the literature about the humidity-sensing applications of Cu_2_O nanoparticles. Therefore, an extensive study is required to understand the role of Cu_2_O nanoparticles in humidity-sensing devices [24,25]. The physical properties—bandgaps, nanostructures, surface-to-volume ratio, etc.—of Cu_2_O nanoparticles are greatly dependent on their particles sizes, shapes, and morphology. The physical and/or chemical properties of Cu_2_O may vary with different ambient conditions that can make it good candidate for humidity-sensing applications [12,26,27,28].

Humidity is a permanent environmental parameter whose measurement and control are required not only for technologies and industries but also for human comfort [2,29]. The precision of air humidity measurement is one of the basic factors for accurate weather forecasting. A close look at humidity and its consequences discloses that low and high humidity both can harm all kinds of living or nonliving things, for example, the growth of fungus on materials in wet conditions on food products, papers, and leathers, various kinds of fabrics, woods, and corrosion of metals due to soaring humidity [30,31]. On a global scale using remotely placed satellites inbuilt sensors, the humidity is measured in terms of percent relative humidity (%RH) for climate monitoring and weather forecast, which detect the amount of water vapor present in the troposphere [32,33]. The humidity sensors made up of polymeric materials can be allocated into two categories: one is the resistive type and other is the capacitive type. The materials showing the variations in dielectric constant, being used for capacitive-type sensors, while in the resistive type, the electrical resistance of polymers decreases with the absorbing water molecules [4]. The capacitive technique is considered as the most favorable for miniaturized humidity sensors [8,34,35].

Herein, Cu_2_O nanoparticles are synthesized and characterized for their potential use in a polymer-based humidity sensor. So, an organic–inorganic hybrid sensor based on a conducting polymer F8 and inorganic semiconductor Cu_2_O nanoparticles is fabricated and investigated for its potential applications as humidity sensor. To take advantage of the hydrophobic nature, stability and large surface-to-volume ratio of Cu_2_O nanoparticles, the humidity-sensing properties of F8 are enhanced by blending F8-Cu_2_O nanoparticles together. The inherent electrical conductivity of F8 alone is not very high, which is insufficient for a high sensitivity of humidity. Therefore, the development of an efficient conductive system between Cu_2_O nanoparticles and the F8 polymer matrix can improve the hole transport rate and increase the humidity sensitivity. The F8-Cu_2_O nanocomposite offers both the characteristics of polymer and inorganic nanomaterial simultaneously. The key mechanism responsible for better sensitivity of the F8-Cu_2_O nanocomposite is the contact between embedded Cu_2_O nanoparticles and the F8 matrix to ensure smooth charge carrier transfer through the interface of F8/Cu_2_O nanoparticles. For this purpose, the Ag/F8-Cu_2_O/Ag sensor is characterized for its humidity-sensing properties by measuring its capacitance as a function of relative humidity (%RH). To study the enhancement, the results obtained here are compared with that of Ag/F8/Ag sensor already reported in the literature.

## 2. Experimental Work

### 2.1. Synthesis of Cu_2_O Nanoparticles

Copper sulfate pentahydrate (CuSO_4_.5H_2_O) having purity >99%, sodium borohydride (NaBH_4_) with purity >97%, polyethylene glycol 6000 (PEG 6000), sodium hydroxide NaOH purity >98%, and ascorbic acid having purity >99%, are used as reagents for the synthesis of Cu_2_O nanoparticles; all these materials are of analytical grade and obtained from Sigma-Aldrich, Darmstadt, Germany. PEG, NaBH_4_, ascorbic acid, and NaOH are employed as the capping reagent, reducing agent, an antioxidant, and pH level adjustment of the solution, respectively.

Initially, a uniform solution is made by magnetically stirring CuSO_4_.5H_2_O in deionized water at 0.025 g/mL concentration, which is followed by dissolving PEG at a concentration of 0.12 g/mL in the same solvent. When later solution is put in former solution, a white solution is achieved. Afterwards, 4 mg/mL and 9 mg/mL concentrated solutions of NaOH and ascorbic acids, respectively, are added to the already prepared solution of CuSO_4_.5H_2_O + PEG. At the end, an aqueous solution of NaBH_4_ at 8 mg/mL is formed and added to the previously prepared solution and kept for stirring to make a uniform solution. Consequently, an aqueous dark reddish-brown solution is formed, which is kept overnight to cool. The next morning, the solution changes its color to yellow, which is centrifuged and, lastly, the precipitate is collected and dried.

### 2.2. Device Fabrication

**Figure 1:**
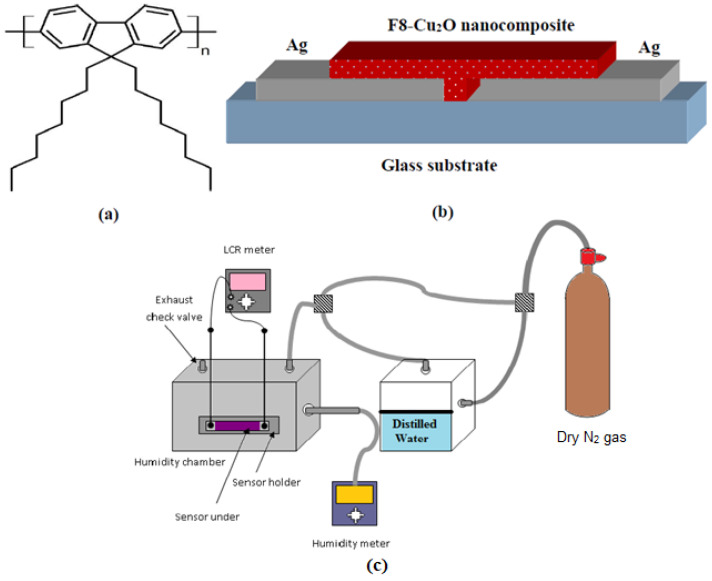
(**a**) Molecular structure of F8, (**b**) Schematic structure of Ag/F8-Cu_2_O/Ag sensor, (**c**) Schematic diagram of self-developed humidity sensing set up.

The conjugated polymer F8 and Ag having 99.9% purity are obtained from Cambridge Display Technology (CDT), UK and Sigma-Aldrich, Darmstadt, Germany, respectively. The molecular formula and molecular mass of F8 is (C_29_H_41_)_n_ and 151.987 g/mol, respectively, whereas its molecular structure is shown in Figure 1a. For the fabrication of the F8-Cu_2_O nanocomposite-based sensor, F8 is used as an active matrix material, while Cu_2_O nanoparticles are used as the dopant, and a commercially available soda lime microscopic glass slide (25 mm × 20 mm) is used as a substrate. Firstly, to remove dust particles and other contaminations from the glass substrate, it is cleaned in acetone and iso-propanol for 10 min using an ultrasonic bath. Then, it is dried by blowing with dry nitrogen (N_2_) gas. Photolithography is used to create an inter-electrode gap of ≈5 μm between Ag electrodes. Afterwards, Ag electrodes are thermally deposited on the photo-lithographically pre-prepared samples by using an EDWARDS AUTO 306 vacuum thermal evaporator at a vacuum level of 1.5 × 10^−5^ mbar. The deposition rate is kept uniform and maintained at 0.1 nm/s. The film thickness was measured by an in-situ FTM5 quartz crystal thickness monitor. For solutions preparation, a 10 wt% uniform solution of F8 is prepared in toluene using a magnetic stirrer for 4 h. Similarly, 1 wt% of uniform dispersion of Cu_2_O nanoparticles is prepared in the same solvent. To make an F8-Cu_2_O nanocomposite, the solutions are physically mixed in 10:1 wt% and magnetically stirred for further 2 h. After making uniform solution of F8-Cu_2_O nanocomposite, it is spin coated on the pre-patterned 5 μm gap between Ag electrodes to form an Ag/F8-Cu_2_O/Ag surface-type sensor. The thickness of the F8-Cu_2_O nanocomposite thin film (≈100 nm) is measured by a Dektak profilometer. The device is left to dry for 5 h at 50 °C and also to release mechanical stresses and strains from the film. The schematic diagram of the fabricated sensors is shown in Figure 1b.

The surface morphology of Cu_2_O nanoparticles and F8-Cu_2_O nanocomposites thin films are carried out by using a TEM Philips SM-12 with 70 μm lens operating at 100 kV with a 2.0 × 10^−10^ point-2-point resolution, SEM JEOL JSM-840 having an electron gun with an operating voltage 0.2 to 40 kV, and an AFM Nanoscope-IIIa SPM from Dimension TM 3100 Digital Instruments Veeco Metrology Group installed with a silicon cantilever having a resonance frequency, force constant, and tip radius of 160 kHz, 5 N/m, and <10 nm, respectively. The ultraviolet-visible (UV–vis) spectrophotometer (PerkinElmer Lambda 1050 UV/vis/NIR spectrometer) is used to record the absorption spectrum of the thin film. The XRD pattern is recorded with a Philips X’Pert X-ray powder diffractometer using 40 kV, 30 mA, Cu Kα radiation (*λ* = 1.54 Å) with a scan speed of 2*θ* = 5°/min in the range from 2*θ* =10°–85°. FTIR spectra are measured with a PerkinElmer Spectrum^™^ 3 FT-IR spectrometer. The humidity-sensing measurements are made on self-developed humidity sensing set up as shown in Figure 1c. The capacitive measurements of the Ag/F8-Cu_2_O/Ag sensor are carried out by placing it in the sealed chamber, as shown in Figure 1c, connected with a digital SERIE P-320 probe hygrometer. The capacitance is measured by an ESCORT ELC-133A LCR meter at 1 kHz and 120 Hz. The level of humidity in the chamber is controlled by the flow of N_2_ gas after passing through the water chamber. All the measurements are carried out at room temperature (25 °C).

## 3. Results and Discussion

### Materials Characterization

**Figure 2:**
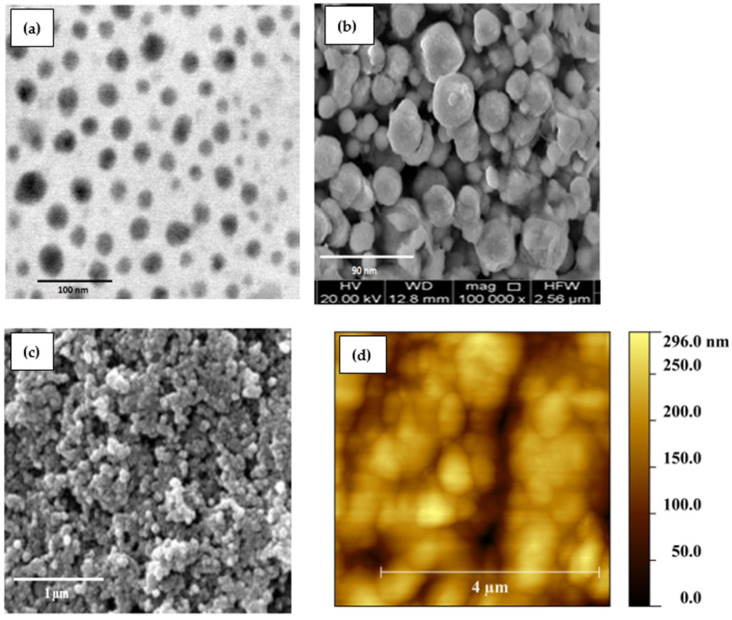
(**a**) TEM and (**b**) SEM images of Cu_2_O nanoparticles; (**c**) SEM and (**d**) AFM images of F8-Cu_2_O nanocomposite thin films.

In Figure 2a, a TEM micrograph of the synthesized Cu_2_O nanoparticles is shown, which exhibits a slightly non-uniform distribution of nanoparticles. Most of the Cu_2_O nanoparticles can be seen, which are individually distributed far from one another, whereas at a few regions, there are bigger sized nanoparticles, which actually show agglomeration and formed mosaics. The agglomeration of these nanoparticles is due to non-uniform and uneven precipitation during synthesis. The average Cu_2_O nanoparticle size is measured to be approximately 24 ± 2 nm. The TEM micrograph verifies the successful synthesis of Cu_2_O nanoparticles. In support of TEM images of the Cu_2_O nanoparticle, its SEM image is also studied to know the surface morphology, observe the nanoparticles’ shapes and sizes, and compare it with the TEM micrographs. The SEM image of the Cu_2_O nanoparticle is shown in Figure 2b, which is in good agreement with the TEM results.

The surface morphology of the active thin films greatly affects the performance of the devices and sensors. Therefore, it is important to know and study the surface features of F8-Cu_2_O nanocomposite thin films. Figure 2c shows the SEM image of the F8-Cu_2_O nanocomposite thin film. The image shows mix phases due to the polymer F8 and Cu_2_O nanocomposites. There can be bunches and an agglomeration of particles which are, in some regions, non-uniform with voids and interstitial spaces between them. However, in some regions, there are almost uniform distributions of F8 and Cu_2_O nanoparticles, which are equally distributed. To correlate the surface morphology of the F8-Cu_2_O nanocomposites film, an AFM image is also measured to confirm the SEM micrograph. Figure 2d shows an AFM image of the F8-Cu_2_O nanocomposites thin film on a scale size of 5×5 µm measured via non-contact mode at a scan rate of 0.85 Hz. The image shows the same morphology and distribution of F8 and Cu_2_O nanoparticles as that of the SEM image. Herein, the nanospheres can be seen, which are Cu_2_O nanoparticles, whereas the contrast represents the F8 film. The value of average surface roughness (R_a_) is about 22 ± 2 nm. Due to this surface roughness, there can be seen some pores and voids at different sites of the image. However, this porosity of the film is utilized as an advantageous tool for the humidity sensor because of the better absorption and desorption of water vapors.

**Figure 3:**
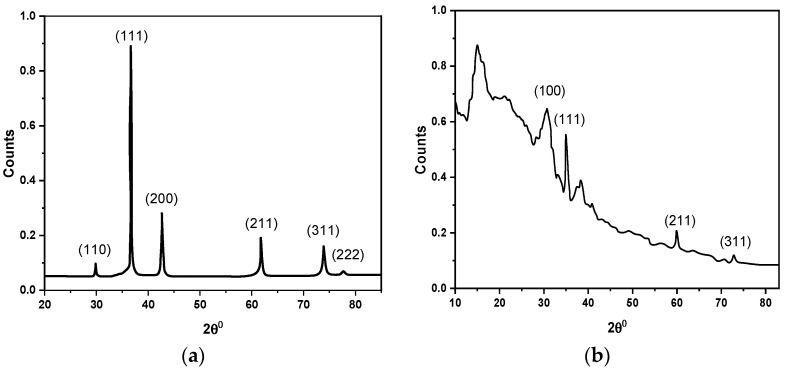
XRD spectra of (**a**) Cu_2_O nanoparticles; (**b**) F8-Cu_2_O nanocomposite.

Figure 3a,b show XRD spectra of the synthesized Cu_2_O nanoparticles and F8-Cu_2_O nanocomposite, respectively. The sharp and intense peaks in Figure 3a confirm the crystalline nature of Cu_2_O with an octahedral structure single phase according to (JCPDS no: 05-0667). The crystallites′ Miller indices (hkl) and two theta (2θ) are compared to those of the same crystallites in powder diffraction file (PDF) files deposited in a database. The (110), (111), (200), (211), (220), (311), and (222) crystal planes of cubic (primitive) Cu_2_O are assigned to the diffraction peaks at 2θ= 29.55, 36.41, 42.29, 52.4, 61.34, 73.53, and 77.5, respectively with (JCPDS 05-0667). No peaks of impurities are observed in the XRD spectrum. The peaks are significantly widened (full width at half maximum), indicating that Cu_2_O is composed of nanocrystals. The highest peak intensity of the (111) plane indicates that Cu_2_O crystals have the larger atomic densities per unit area of this plane. On the other hand, the peaks at (100), (111), (311), and (311) are associated with diffraction peaks at 2θ= 30, 35, 60, and 73.5, respectively. The XRD results of the F8-Cu_2_O nanocomposite indicate a few peaks, which are due to the presence of Cu_2_O nanoparticles.

**Figure 4:**
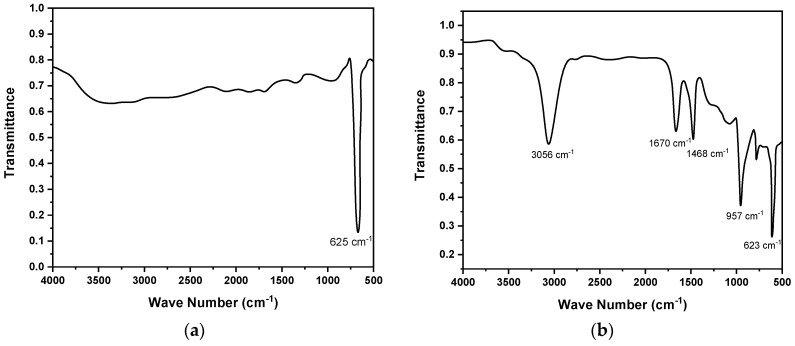
FTIR spectra of (**a**) Cu_2_O nanoparticles and (**b**) F8-Cu_2_O nanocomposite.

The FTIR spectra of Cu_2_O nanoparticles and F8-Cu_2_O nanocomposites are shown in Figure 4a,b, respectively. The spectra are measured in the energy range from 500 to 4000 cm^−1^ where wavenumber vs. percent transmittance (%T) is plotted. The common strong peaks observed in both spectra at 623 and 625 cm^−1^ indicate the vibration mode of Cu_2_O. The broad absorption peak at 957 cm^−1^ is due to the C-H out-of-plane bending mode. The energy bands and their assigned bond dynamics observed in the FTIR spectra are presented in Table 1. The modes of vibration assigned to 957 and 1408 cm^−1^ represent the out-of-plane bending and CH_2_bending mode, respectively. The C=C aromatic ring of benzene stretches are associated with the peak observed at 1670 cm^−1^. The peaks witnessed at 3056 cm^−1^ identify a C-H stretch of π conjugation. Therefore, all the indicated peaks and bands observed confirm the chemical composition of the Cu_2_O and F8-Cu_2_O nanocomposite.

**Figure 5:**
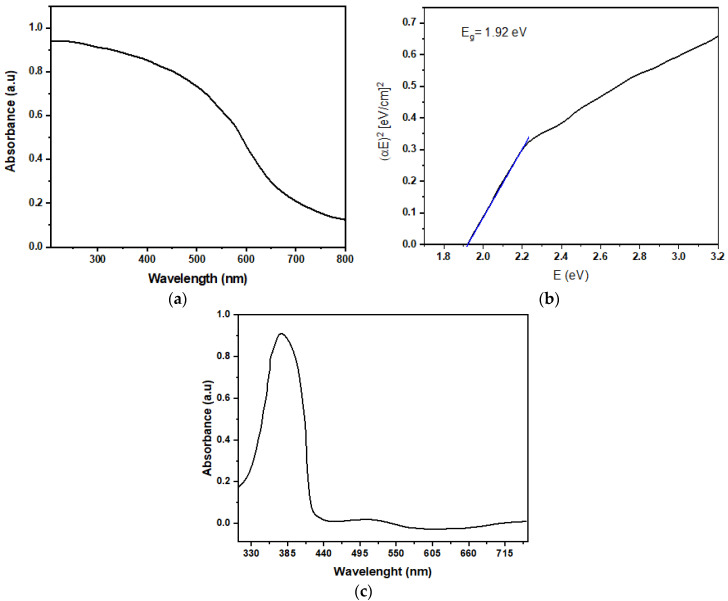
(**a**) UV-Vis spectrum and (**b**) Tauc’s plot of Cu_2_O nanoparticles (**c**) UV-vis spectrum of F8 film.

The optical bandgap (E_g_) of Cu_2_O and F8-Cu_2_O nanocomposites is calculated by applying Tauc’s Equations (1) and (2) on their corresponding UV-Vis spectra, as shown in Figure 5a–c. From Figure 5a, Cu_2_O appears to absorb visible light over a broad wavelength from 300 to 650 nm. The shape, size, and crystallinity of Cu_2_O crystals have a significant impact on UV-vis absorption. Therefore, the size and crystallinity of Cu_2_O octahedral crystals can be linked to the blue shift in the spectrum where the crystallite is smaller than 50 nm, which is herein approximately 24 nm. Figure 5b shows the Tauc’s plot of Cu_2_O nanoparticles, which describes the value of E_g_ to be 1.92eV, which is in close agreement with that reported in the literature.

For calculating the value of E_g_, the following Tauc’s equations are applied:

αE = (E − E_g_)^m^ (1)

where α, E, E_g_, and m are the absorption coefficient, energy of incident photon, bandgap of material, and direct or indirect transitions constant, which is m = ½ for direct transition and 2 for indirect transition, respectively. The value of α is determined from Beer–Lambert law that is given as follows:

*A* = *ln*(*I_0_*/*I*) = *αd* (2)

where *A* is the absorbance, *I_0_* is the incident intensity of light, *I* is the transmitted beam intensity, and *d* is the thickness of the film.

Among the ambient environmental conditions, humidity is an important parameter due to its effects on human comfort and industries. Important industries in this context are medicine and food processing, agriculture, storage, etc. The relative humidity is expressed as:

(3) $$\%\mathrm{RH}=\frac{\mathrm{actual}\;\mathrm{vapor}\;\mathrm{density}}{\mathrm{saturated}\;\mathrm{vapor}\;\mathrm{density}}\times 100$$

**Figure 6:**
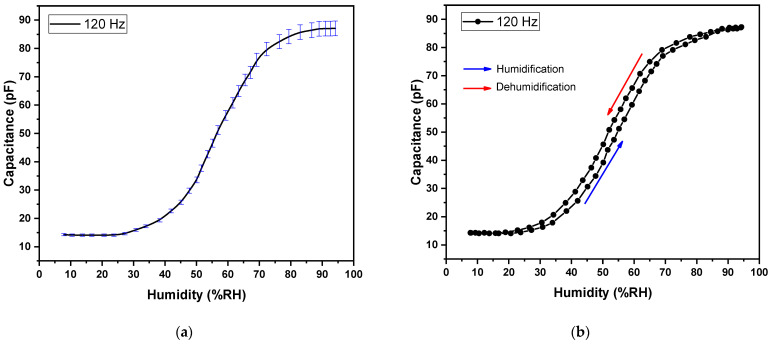
(**a**) Capacitance vs. relative humidity curve and (**b**) Hysteresis loop of Capacitance vs. relative humidity for Ag/F8-Cu_2_O/Ag sensor at 120 Hz.

**Figure 7:**
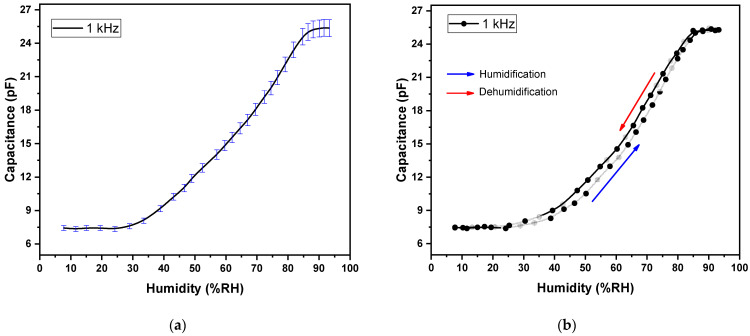
(**a**) Capacitance vs. relative humidity curve and (**b**) Hysteresis loop of Capacitance vs. relative humidity for Ag/F8-Cu_2_O/Ag sensor at 1 kHz.

The effect of humidity on a device can be measured as a change in capacitance (C). The C-%RH response of Ag/F8-Cu_2_O/Ag device (a surface-type humidity sensor) is shown in Figure 6a,b. In response to %RH in the range of 8–95%RH, the capacitance increased from 0.012 to 0.282 nF (23.5 times) at 120 Hz frequency at room temperature. This increase is much better than our previous work on F8 [16] and F8-CdSe quantum dots-based sensors [4]. Thus, the doping of Cu_2_O nanoparticles improved the sensing characteristics of F8. Similarly, at 1 kHz and room temperature, the plot of %RH vs. C of the device is shown in Figure 7a. In this case, the capacitance increases from 12.3 to 72.1 pF (5.86 times) as the %RH varies from 8 to 95%RH. Here again, it increases more than our previous work on F8 and F8-CdSe quantum dots-based sensors [4,16]. Comparing the humidity sensing at both frequencies, i.e., 120 Hz and 1 kHz, shows that the Ag/F8-Cu_2_O/Ag sensor performs well at lower frequency (120 Hz).

The fabricated sensor Ag/F8-Cu_2_O/Ag is a coplanar device analogous to a parallel plate capacitor with an active film material (F8-Cu_2_O) as the dielectric. The capacitance of such a kind of device is given as:

*C* = *AƐ_0_*/*d* (4)

*Ɛ_0_* is the permittivity of free space, *Ɛ_r_* is the relative permittivity of the material between plates of the capacitor, which is the range 4–8 for organic semiconductors [36], *A* (effective area) and *d* (separation between the plates, 5 µm). Water (H_2_O) is a polar molecule, and it has the highest dielectric constant, i.e., 80 [37]. The rough surface and porous surface morphology of F8-Cu_2_Onanocompositefilm capture water molecules, which in turn increases the effective dielectric constant of the active film and, hence, the capacitance of the Ag/F8-Cu_2_O/Ag device increases, according to Equation (4). In addition, as F8 and Cu_2_O both are p-type, the conductivity of the F8-Cu_2_O nanocomposite is generally found out by the hole carrier density of the inter-grain boundary contact regions. When the Ag/F8-Cu_2_O/Ag sensor is exposed to the water vapors, they are adsorbed onto the surface of the F8-Cu_2_O film. The adsorbed water molecules at the surface of the F8-Cu_2_O layer—that also contain oxygen—take free electrons from the conduction band of Cu_2_O nanoparticles to produce a chemisorbed oxygen group (O_2_^−^,O_2_^2−^, and O^−^) because of strong electronegativity, which in turn increases the capacitance of the sensor. So, in first place, the adsorption of water molecules takes place at the surface of F8-Cu_2_O film, and an adsorption complex is developed. This process is called chemisorption. Further adsorption of water vapors facilitates the adsorption complex that forms bonds with the other water molecules on the surface of the F8-Cu_2_O layer through hydroxyl groups and, therefore, water molecules become adsorbed on the surface through hydrogen bonding. This phenomenon is known as physisorption. Once the F8-Cu_2_O nanocomposite layer comes into contact with water vapors, the chemisorption process starts, and the water vapors chemisorb on theavailable sites of the F8-Cu_2_O film. Initially, the chemisorbed layer is produced due to the dissociative process and, subsequently, the physisorbed layer is formed. The electrons are accumulated at the surface of the F8-Cu_2_O layer. This, as a result, contributes to increasing the capacitance of the sensor with an increase in %RH. So, the Cu_2_O crystal facet, size and shape, surface area, and heterogeneous interface contact of F8-Cu_2_O are of significant importance in humidity sensing. For better humidity sensitivity, the surface accessibility of F8-Cu_2_O active film is essential to be available to the water vapors. Particularly, nanospheres of Cu_2_O crystals are more suitable for humidity-sensing applications due to their uniform and porous morphology with the F8 matrix.

The graphs shown in Figure 6a and Figure 7a show that the change in capacitance of the sensor is negligible in the range 8–27%RH due to the low amount of water molecules and then starts to increase up to 90%RH, and after this point, there is no change in capacitance. The saturation at higher humidity shows the fact that almost all the available sites/pores in the F8-Cu_2_O nanocomposite film become filled by water vapors and thus result in no more sensitivity after the saturation point. Figure 6b and Figure 7b show a smaller gap in the hysteresis loops of the sensor, which means that the sensor has the potential to respond to humidity in both forward and reverse directions. Since water is a polar molecule [37], the molecules align themselves with the external electric field. This alignment increases the polarizability of the material according to the equation:

(5) P=nαE 

here, α is the polarizability of the material which increases with the polarization *P* (dipole moment per unit volume) and *n* is the number of molecules. In turn, this increase in polarizability affects the capacitance of the device.

**Figure 8:**
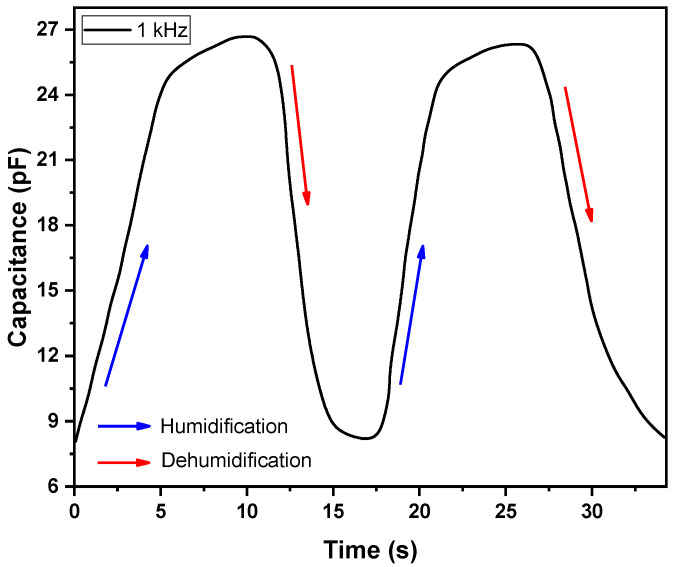
Response time and recovery time cycles of the Ag/F8-Cu_2_O/Ag sensor at 1 kHz.

Response and recovery times are very important in the evaluation of a sensor’s performance. The response and recovery time means the degree of quickness of the sensor toward variation in ambient %RH. During measurements, the device was suddenly moved from a chamber having 8%RH into another chamber having 95%RH by allowing water vapors to enter into the chamber, and the response time was recorded. Similarly, the sensor was then quickly exposed to another chamber which was dehumidified (8%RH) with the help of dry nitrogen, and its recovery time was recorded. The response and recovery curves of the device are shown in the Figure 8. The measured values for the response and recovery times are 9 s and 7s, respectively. The overall performance and comparison of the Ag/F8-Cu_2_O/Ag sensor with previous works, in terms of different humidity sensing parameters, is presented in Table 2.

## 4. Conclusions

Nanoparticles of the Cu_2_O semiconductor are successfully synthesized by the wet chemical route method with an average particle size of 24 ± 2 nm. The TEM and SEM micrographs confirmed the spherical shapes, nearly uniform distribution and size of Cu_2_O nanoparticles. XRD and FTIR spectra verified the single phase crystalline octahedral structure and composition of Cu_2_O nanoparticles, respectively, whereas, their UV-vis curve exhibited a broad absorption spectrum with an optical bandgap of 1.92 eV. The Ag/F8-Cu_2_O/Ag surface type capacitive sensor was prepared by using photolithography and spin-coating techniques. The capacitance vs. %RH of measurements of the device, at 120 Hz and 1 kHz, revealed its sensitivity between a broad range of humidity levels from 27 to 86%RH. However, at 120 Hz, the ratio of C/C_0_ (final capacitance to initial capacitance) of the sensor is 23.5, while at 1 kHz, it is 5.86, which means that the Ag/F8-Cu_2_O/Ag sensor performs well at a lower frequency of 120 Hz. The small gaps between hysteresis loops of capacitance vs. %RH curves of the sensor showed good repeatability and reproducibility measurements of the sensor. The response and recovery times of the sensor were measured to be 9 s and 7 s, respectively. The overall sensitivity of the Ag/F8-Cu_2_O/Ag sensor is better than that reported for F8-based sensors. The better sensitivity of the F8-Cu_2_O nanocomposite is attributed to the hydrophobic nature and also to the Cu_2_O nanocrystals’ large accessible surfaces with the suitable structural properties, which are highly desirable for enhancing humidity sensing. The porous morphological structure of F8-Cu_2_O films is the efficient mechanism that lead to enhancing the sensitivity by capturing and/or accommodating a sufficient amount of water vapors.

## Figures and Tables

**Table 1:** Bond dynamics in Cu_2_O and F8-Cu_2_O thin films.

Energy Bands (cm^−1^)	Bonds Nature/Dynamics
623–625	Cu_2_O Vibration Mode
957	C-H Out-of-Plane Bending
1468	CH_2_Bending
1670	C=C Aromatic Ring Stretch
3056	C-H StretchingSP^2^Hybridization

**Table 2:** Comparison of the humidity sensor’s parameters with previous works.

Humidity Sensor	Frequency (Hz)	Sensing Range (% RH)	Ratio of C/C_0_	Response Time (s)	Recovery Time (s)	Ref.
Ag/F8/Ag	120	30–75	4.0	15	7	[8]
	1000	50–80	1.2	-	-	
Ag/F8:CdSe QDs/Ag	120	25–91	7.3	9	7	[4]
	1000	20–90	2.1	-	-	
Au/N-BuHHPDI/Au	100	0–90	13	60	70	[2]
Ag/F8-Cu_2_O/Ag	120	8–95	23.5	-	-	Present work
	1000	8–95	5.8	9	7	

## Data Availability

Not applicable.
